# Intraoperative spectroscopic evaluation of sentinel lymph nodes in breast cancer surgery

**DOI:** 10.1007/s10549-024-07349-z

**Published:** 2024-05-20

**Authors:** Surekha Barkur, Radu A. Boitor, Raluca Mihai, Navarasi S. Raja Gopal, Samuel Leeney, Alexey A. Koloydenko, Hazem Khout, Emad Rakha, Ioan Notingher

**Affiliations:** 1 School of Physics and Astronomy, University of Nottingham, Nottingham, UK; 2 Department of Pathology, Nottingham University Hospitals NHS Trust, Nottingham, UK; 3 Mathematics Department, Royal Holloway, University of London, London, UK; 4 Breast Institute, Nottingham University Hospitals NHS Trust, Nottingham, UK

**Keywords:** Raman spectroscopy, Auto-fluorescence imaging, Spectral imaging, Lymph node metastasis, Breast cancer

## Abstract

**Background and objectives:**

Sentinel lymph node (SLN) biopsy is a standard procedure for patients with breast cancer and normal axilla on imaging. Positive SLNs on histological examination can lead to a subsequent surgery for axillary lymph node clearance (ALNC). Here we report a non-destructive technique based on autofluorescence (AF) imaging and Raman spectroscopy for intra-operative assessment of SLNs excised in breast cancer surgery.

**Methods:**

A microscope integrating AF imaging and Raman spectroscopy modules was built to allow scanning of lymph node biopsy samples. During AF-Raman measurements, AF imaging determined optimal sampling locations for Raman spectroscopy measurements. After optimisation of the AF image analysis and training of classification models based on data from 85 samples, the AF-Raman technique was tested on an independent set of 81 lymph nodes comprising 58 fixed and 23 fresh specimens. The sensitivity and specificity of AF-Raman were calculated using post-operative histology as a standard of reference.

**Results:**

The independent test set contained 66 negative lymph nodes and 15 positive lymph nodes according to the reference standard, collected from 78 patients. For this set of specimens, the area under the receiver operating characteristic (ROC) curve for the AF-Raman technique was 0.93 [0.83–0.98]. AF-Raman was then operated in a regime that maximised detection specificity, producing a 94% detection accuracy: 80% sensitivity and 97% specificity. The main confounders for SLN metastasis were areas rich in histiocytes clusters, for which only few Raman spectra had been included in the training dataset.

**Discussion:**

This preliminary study indicates that with further development and extension of the training dataset by inclusion of additional Raman spectra of histiocytes clusters and capsule, the AF-Raman may become a promising technique for intra-operative assessment of SLNs. Intra-operative detection of positive biopsies could avoid second surgery for axillary clearance.

**Supplementary Information:**

The online version contains supplementary material available at 10.1007/s10549-024-07349-z.

## Introduction

Breast cancer (BC) is the most frequent cancer among women, with 55,000 new cases diagnosed annually in the UK and 2.2 million worldwide [1]. Because sentinel lymph nodes (SLNs) are the first nodes to be involved when BC metastasizes [2], it is common practice for BC patients without preoperative diagnosis of positive lymph nodes to have SLN sampling rather than axillary lymph node clearance (ALNC) [3–6]. Negative SLN diagnosis eliminates the need for ALNC, which is associated with increased morbidity such as lymphedema, shoulder dysfunction, injury to axillary vein and motor nerves. Although many countries are considering more conservative axillary approaches that avoid ALNC in cases with axillary metastases confined to 1–2 SLNs [7], this practice is not implemented in all centres. Furthermore, it is not applicable to patients not fulfilling the criteria of the trials demonstrating this finding (ACOSOG Z0011, IBCSG 23–01, AMAROS), including patient age, receptor status and the plan to offer local and systemic therapy in addition to patients who were offered neoadjuvant treatment or planned for mastectomy [8–10].

Considering these, positive SLNs confirmed by histological examination (which takes about 1–2 weeks) are often followed by a second axillary operation to evaluate the status of other axillary nodes. SLNs are found positive on histopathological examination in 20–30% of cases [11, 12]. In these cases, non-SLN can be involved in around 40% of cases [12], with involvement of level III lymph nodes in around 10% of cases [13].

Several techniques have been developed for assessing SLNs intra-operatively [14]. Frozen section histology and imprint cytology are fast and technically inexpensive but have relatively low sensitivities (44–96%) and require the availability of experienced pathologists [15–17]. Touch imprint cytology also requires expertise to be present onsite, which is impractical in many places [18, 19]. One-step nucleic acid amplification (OSNA) can be used intra-operatively (typically within 30–45 min) to detect cytokeratin-19 (CK19). A meta-analysis study indicated a sensitivity and specificity of 87% (95% confidence intervals (CI) 81–93%) and 98% (CI 96–100%) respectively [20], which were confirmed by a second study reporting pooled sensitivity and specificity of 87% (CI 81–91%) and 92% (CI 86–95%) respectively [21]. However, one of the main limitations of OSNA is that it consumes the tissue and thus compromises histology evaluation of the excised nodes. Reserving half of the tissue for histology would provide a reference but leads to allocation bias. In addition to the fact that up to 7% of BC do not express CK19 [22, 23], other CK19 positive tumours may involve the axillary nodes, and these cannot be determined, as the tissue is consumed in the molecular testing. Metasin test (detects CK19 and mammaglobin) needs molecular biology experts to be present onsite, in addition to similar concerns as that for OSNA [11].

Raman spectroscopy is an optical technique that measures the intrinsic molecular properties of tissue and can be used for medical diagnosis [24, 25]. Initial studies on lymph node biopsies indicated 80–90% sensitivity and 85–100% specificity for discriminating between metastasis and normal lymphoid tissue [26–28]. While these studies highlighted the potential of Raman spectroscopy for intra-operative assessment of LNs, measuring whole LNs by raster scanning was impractical because of long acquisition times (several hours). Recently, selective-sampling Raman microscopy techniques have been reported that can reduce acquisition times using real-time computational or faster optical imaging techniques to guide Raman spectroscopy measurements [29–31].

In this study, we optimised a selective-sampling technique based on a confocal AF imaging (405 nm laser excitation) and Raman spectroscopy (785 nm laser excitation) to evaluate its feasibility of detecting positive SLNs resected during BC surgery.

## Patients and methods

### Patients and tissue samples

All lymph node samples (LNs) were obtained from patients undergoing breast cancer surgery at Nottingham University Hospitals National Health Service (NHS) Trust. Ethical approval was obtained from East Midlands—Leicester South Research Ethics Committee (19/EM/0251). LNs were measured either fresh or after short time fixation in formaldehyde (10% formalin). The AF-Raman technique was tested on a set of 81 lymph nodes collected from 78 patients recruited randomly (details on sample size calculation and estimates of confidence intervals included in the Supplementary Information).

None of the recruited patients were treated with neoadjuvant chemotherapy. The set was comprised 58 fixed and 23 fresh specimens. Nodes with a diameter less than 1 cm in size were bisected whereas nodes larger than 1 cm were cut into 2 mm thick layers. These samples were placed on a quartz window (2.5 cm × 5 cm, 1 mm thickness) for AF and Raman spectroscopy measurements. After the AF-Raman measurements, 10 µm thick tissue sections were cut from the investigated LN surface and stained by hematoxylin and eosin (H and E) for post-operative histology. A histological assessment was performed for each lymph node included in the study, whilst blinded to the results produced by AF-Raman.

### Instrumentation

**Fig. 1:**
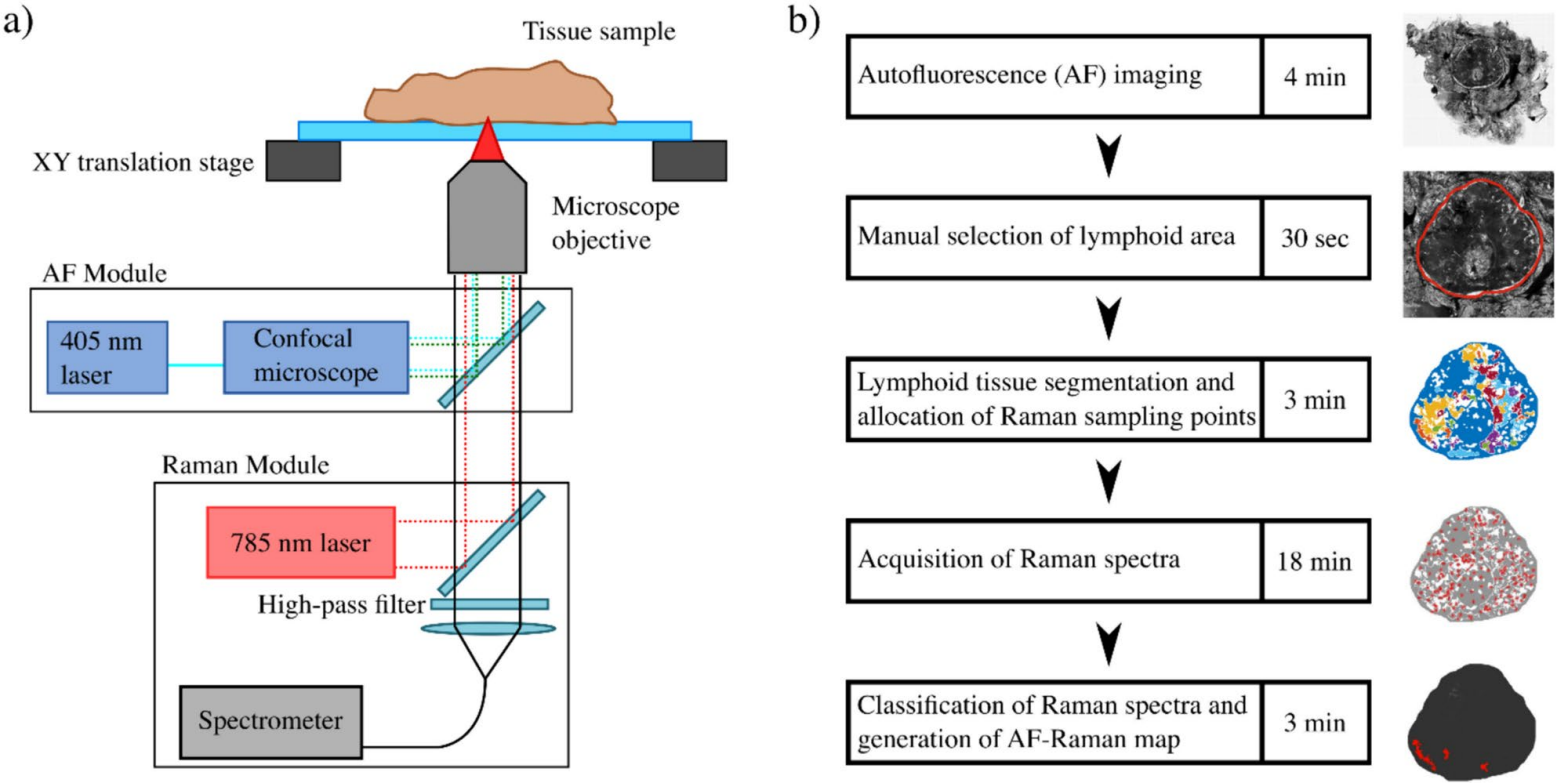
Schematic description of the AF-Raman instrument, measurement, and analysis workflow: **a** Schematic representation of the AF-Raman instrument; **b** AF-Raman measurement workflow, producing an AF-Raman map in less than 30 min. Detected metastasis areas are represented in red

The instrument consisted of an inverted optical microscope (Nikon eclipse Ti) equipped with an automated sample stage (H107 with Proscan II controller, Prior Scientific), a confocal fluorescence module (Nikon C2) and a Raman spectroscopy module (Fig. 1a). The AF module included a 405 nm laser (Coherent, Obis 405) and a photomultiplier tube for detection. AF images were recorded using a range of microscope objectives (2 × to 10x, Nikon) and by moving the microscope translation stage laterally to get image tiles, which were then stitched together into a single image of the sample.

For Raman spectroscopy, a 785 nm laser (Toptica, XTRA) was focused with a 60x/0.9 NA oil immersion objective. Laser power at the sample was ~ 120 mW. Back scattered light from the microscope objective was focussed onto an optical fibre connected to a spectrometer (77,200, Oriel, Newport, with a 1000 lines/mm ruled diffraction grating) equipped with a cooled back illuminated deep depletion CCD (DU401A Andor Technology).

### Raman spectroscopy classification model

As Raman spectra were collected prior to H&E staining, auto-fluorescence (AF) images were used to guide Raman spectra collection as raster scans for the classification model spectral database (20 or 30 µm steps, 3 s/pixel integration time). Up to seven areas of the sample (each area being 0.4 × 0.4 mm^2^ to 1 × 1 mm^2^ in size) were measured to increase the probability of capturing spectra from metastasis regions. To avoid tissue alteration during the raster scanning, the Raman spectra used to train the classification model were recorded from LNs fixed in formaldehyde. A database of Raman spectra was acquired, with a total of 4571 spectra from 60 patients: 3955 from normal lymphoid tissue and 616 from metastasis. The annotation of the Raman spectra according to tissue type was made by comparing the AF images to the H&E-stained histology sections obtained after histology. The processing of Raman spectra is detailed in the Supplementary Information. After spectral annotation, Raman spectral features were extracted from each spectrum as the area under selected Raman bands. These features were used to train a set of classification models. A total of 42 Raman bands were selected, which were then vector normalised to unity per spectrum.

Spectral classification was performed as a two-step process. A two-class linear discriminant analysis (LDA) model was first utilised to discriminate between adipose and non-adipose tissue. The spectra classified as non-adipose tissue were then classified by a second classification model aiming to discriminate metastatic tissue from other normal lymphoid tissue structures. Several classification techniques were utilised: LDA, multinomial logistic regression (MNLR), random forest, k-NN, SVM and artificial neural network (ANN). A subset of 10 spectral features was selected by retaining the features that showed the highest discriminant power in a single-feature t-test between the metastasis class and all other classes combined (Table S1). For each classification model, the combination of features from this subset that produced maximum specificity in a fivefold cross-validation with sensitivity constrained to be at least 90% was retained for the integrated AF-Raman analysis.

### Integrated AF-Raman analysis

The combined AF-Raman measurements consisted of the following steps (Fig. 1b): AF imaging, manual selection by the user of the LN area in the AF image, automated segmentation and generation of sampling points for Raman spectroscopy, acquisition of Raman spectra, classification of the Raman spectra, and generation of the final diagnosis image by labelling each segment based on the Raman classification results.

Because in standard LN biopsies the LN is surrounded by adipose tissue, the LN was selected manually using the capsule as guidance (rich in collagen, thus appearing bright in the AF images) – this generated a binary mask eliminating the adipose tissue from any further analysis. The remaining LN in the AF image was then segmented using the Canny edge detection algorithm (described in detail in Supplementary Information). Sampling points for Raman spectroscopy were distributed in all segments, and then Raman spectra were acquired at these locations (5 s exposure time). To keep the total AF-Raman measurement time below 30 min, the number of Raman spectra per sample was capped to 200, with a minimum of two sampling points allocated to each segment. If the number of segments was less than 100, sampling points were distributed based on the area of the segments and the intra-segment variance of the AF intensity.

The final AF-Raman diagnosis image of the tissue surface was generated by labelling each segment independently. A segment was labelled as metastatic if more than 50% of spectra within the segment were classified as metastatic and the average spectrum within the segment was also classified as metastatic. The AF-Raman maps were compared to the post-operative histological reports to determine the sensitivity and specificity of the technology after the completion of AF-Raman measurements. The 95% confidence intervals (CI) for sensitivity and specificity metrics were calculated using the Clopper-Pearson intervals. The receiver operating characteristic (ROC) curve was calculated by adjusting the per segment detection threshold for the AF-Raman analysis and computing the sensitivity and specificity for each threshold. The 95% confidence intervals (CI) for the area under the ROC curve metrics were calculated using the bootstrap sampling option of the native Matlab function *perfcurve*, with the number of bootstrap replicas set to 1000.

## Results

**Fig. 2:**
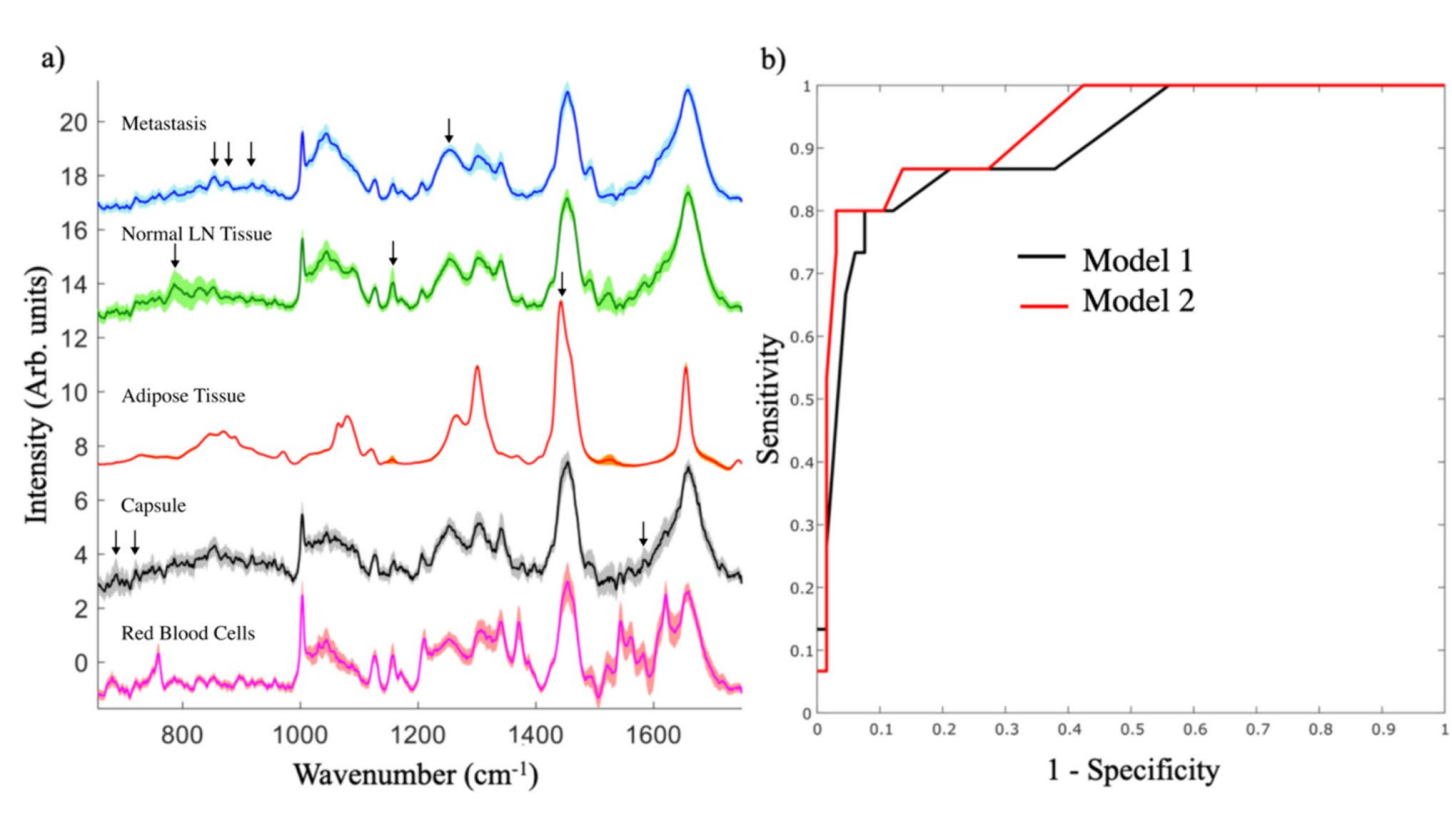
**a** Average Raman spectra of metastasis and normal lymphoid tissue structures (LN). Spectra are shifted vertically for clarity. The shading indicates the standard deviation of each individual component of the spectra. Black arrows indicate the 10 spectral features identified to produce the best discrimination between metastasis and other tissue structures. The arrows are placed above the spectra that showed the highest intensity of each feature. **b** Receiver operating characteristic (ROC) curve for the combined AF-Raman spectroscopy tests: SLN positive Yes/No (histology was standard of reference). Model 1: SLN positive if at least one segment classified as metastasis; Model 2: SLN positive if at least one segment larger than 350 µm or two or more segments (regardless of size) were classified as metastasis. Area under the ROC curve (AUC): Model 1 0.90 [0.79–0.97]; Model 2 0.93 [0.83–0.98]

The average Raman spectra of metastasis and normal lymphoid tissue are presented in Fig. 2a. A single-feature t-test was used to identify the Raman bands that showed the most significant differences between metastatic and normal/benign tissue. Compared to the Raman bands of normal lymphoid tissue, the spectra of metastasis were found to have higher intensity bands at 850 cm^−1^ (proteins, ring breathing in Tyr, collagen C–C stretching), 873 cm^−1^ (collagen hydroxyproline, perhaps overlap with C–N antisymmetric stretching vibrations of choline head group), 945 cm^−1^ (Proteins, C–C backbone stretching), and lower intensity bands at 698 cm^−1^ (C-S bond stretching in methionine, 718 cm^−1^ (phospholipids, C-N symmetric stretching of the choline head group), 784 cm^−1^ (DNA/RNA O–P–O symmetric stretch and ring breathing in uracil, cytosine, thymine), 1338 cm^−1^ (proteins Cα-H deformation), and 1450 cm^−1^ (CH_2_ scissoring and deformations in proteins and lipids), and 1584 cm^−1^ (DNA/RNA guanine, adenine).

Multiple classification models were trained with subsets of the 10 spectral bands identified by the single-feature t-test analysis. Cross-validation results for the best performing models at discriminating between metastatic and normal lymphoid tissue are presented in Table S2. At a target sensitivity of ~ 90%, all classification models delivered > 80% sensitivity and > 80% specificity, with the highest performance achieved by a KNN model: 87.1 ± 0.4% sensitivity and 86.4 ± 0.3% specificity.

After optimisation of the algorithms (see Supplementary Information), the AF-Raman instrument, with all hardware and software parameters locked, was tested on 81 LNs from 78 new patients (58 samples fixed in formaldehyde and 23 fresh samples). The dataset contained 15 positive (12 fixed and 3 fresh) and 66 negative samples (46 fixed and 20 fresh). Patient and disease information for the positive lymph nodes is included in the Supplementary Information Table S3. The Raman spectral differences between fixed and fresh LN metastasis are presented in Supplementary Information.

Two diagnosis models were tested for determining whether a SLN sample was positive or not. For Model 1, a SLN was diagnosed as positive if at least one segment was classified as metastasis. For Model 2, the SLN was classified as positive if either at least one segment larger than 350 µm was classified as metastasis or two (or more) segments regardless of size were classified as metastasis. Using histology as the standard of reference, the receiver operating characteristic (ROC) curves for the two diagnostic models are presented in Fig. 2b. For Model 1, the area under the curve (AUC) was 0.90 [0.79–0.97]. One example of a clinically relevant regime prioritizing specificity (to minimise false positives) indicated 92.42% specificity [95% CI 91.24–93.15] and 80% sensitivity [95% CI 75.38–83.16] (12 true positives, 61 true negatives, 3 false negative and 5 false positives). When a threshold of 350 µm was set on the size of the smallest segment to be considered metastasis (Model 2), the area under the curve (AUC) was 0.93 [0.83–0.98]. For the same level of 80% sensitivity, this model achieved 96.97% specificity [95% CI 95.82–97.59] (12 true positives, 64 true negatives, 3 false negative and 2 false positives).

**Fig. 3:**
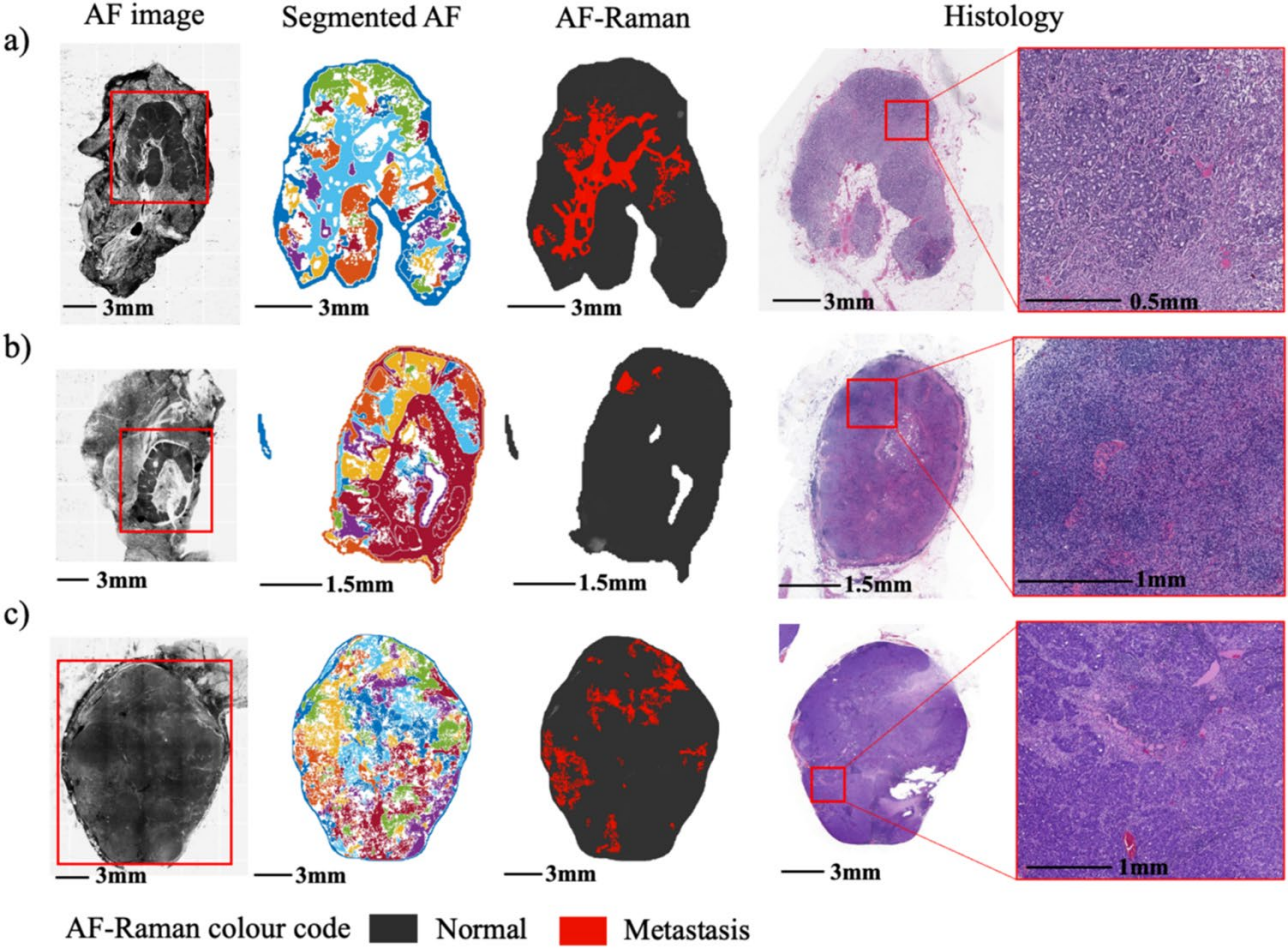
Representative examples of true positive AF-Raman results (Model 2 operating regime: 96.97% specificity). The metastatic regions are presented in red in the AF-Raman images. The histology images are presented for comparison

Figure 3 presents representative cases of true positive SLNs detected by Model 2 when operating in the diagnosis regime of 96.97% specificity and 80% sensitivity. The diameter of the metastatic areas detected via AF-Raman ranged from > 6 mm (as in Fig. 3a) to ~ 1–2 mm (as in Fig. 3b–c). Larger metastatic areas, such as the one shown in Fig. 3a, often had multiple segments classified as positive over the area corresponding to the metastasis.

**Fig. 4:**
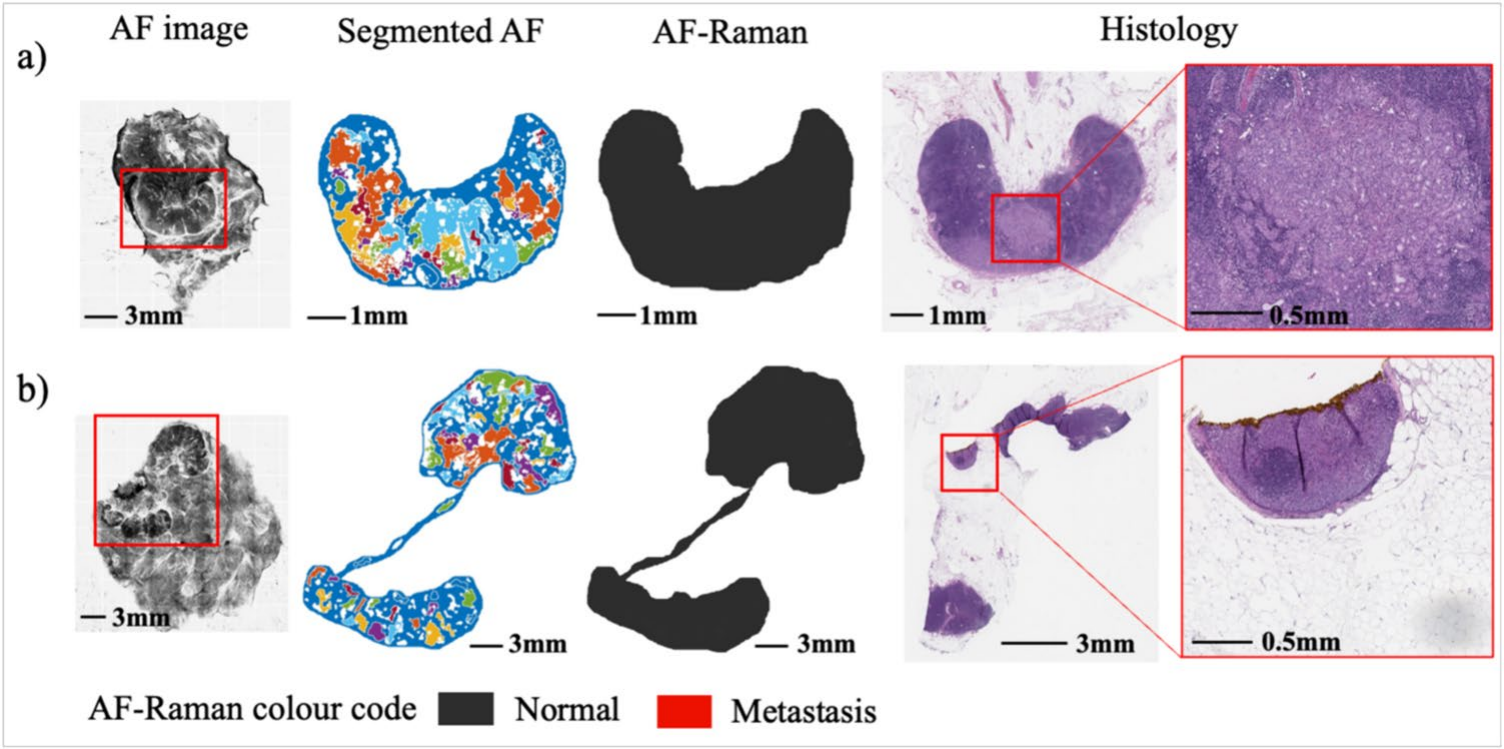
The two false negative cases of AF-Raman diagnoses (Model 2 operating in 96.97% specificity regime). Histology images are presented for comparison, including magnified extracts of possible metastasis confounding regions

Figure 4 shows the two samples for which both models provided a false negative diagnosis when operating in the 92.42% (Model 1) or 96.97% (Model 2) specificity regimes. Based on the histology H&E-stained sections, the size of the metastasis areas were ~ 1.7 mm for sample a) and ~ 0.5 mm for sample b).

**Fig. 5:**
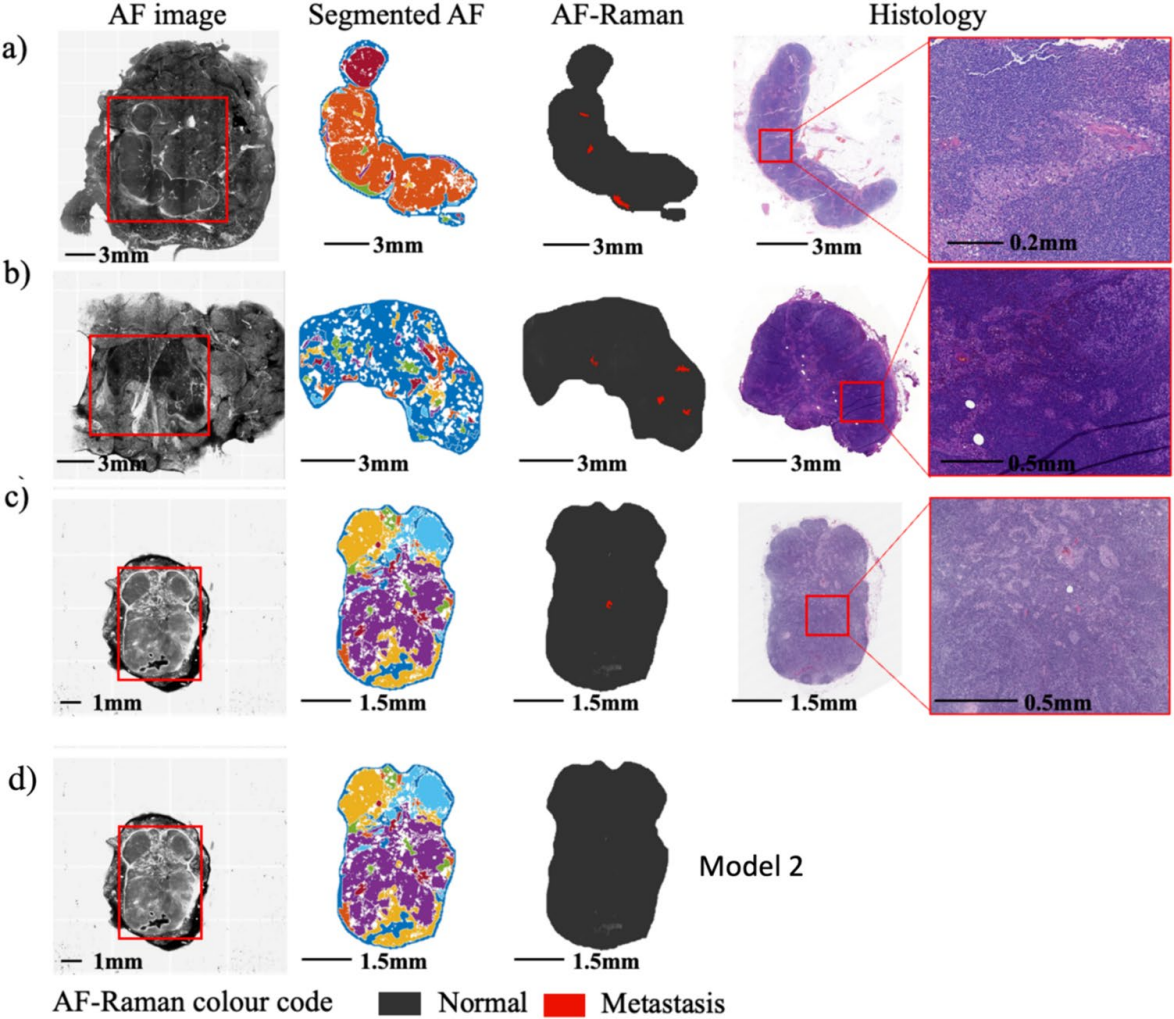
False positive AF-Raman results. **a**–**c**) using Model 1 (regime 92.42% specificity, 80% sensitivity). **d** diagnosis map for sample in **c** using Model 2 (96.97% specificity, 80% sensitivity)

Three false positive samples from Model 1 (regime 92.42% specificity, 80% sensitivity) are presented in Fig. 5(a–c). By comparing the AF-Raman diagnosis maps to the histology sections, the false positive segments appear located inside the LN and likely corresponded to histiocytes. For the typical example in Fig. 5c, a single segment (~ 250 µm in diameter) resulted in a positive classification by the AF-Raman analysis. For this sample, the diagnosis model that included a size threshold of 350 µm (Model 2) provided a true negative diagnosis (Fig. 5d).

## Discussion

The main objective of this study was to investigate whether a dual-modality technique combining AF imaging and Raman spectroscopy could be used to detect metastatic lymph nodes within timescales compatible with intra-operative use. SLN biopsy is widely accepted as the preferred procedure for identifying lymph node metastasis. However, re-excision of the axillary tissue following histological confirmation of positive SLN is a major disadvantage of this approach. Non-destructive techniques that can detect positive SLNs intra-operatively would overcome the limitations and provide a cost-efficient tool to assess SLN intraoperatively without consuming the nodal tissue.

This study reports the first investigation of an integrated AF-Raman spectroscopy technique to detect positive LNs during breast cancer surgery. After training and optimising the measurement and analysis algorithms, independent testing on 81 LN samples (15 positive, 66 negatives) indicated 92.42% specificity [95% CI 91.24–93.15%] and 80% sensitivity [95% CI 75.38–83.16%] (12 true positives, 61 true negatives, 3 false negative and 5 false positives) (Model 1). No link was observed between tissue processing procedure (fixed or fresh) and incorrect AF-Raman assessments. Of the 3 false negative cases, 2 were produced on fixed specimens and 1 on a fresh specimen. Of the 5 false positive cases, 3 were produced on fixed specimens and 2 on fresh specimens. Because current guidelines indicate different approach on ALNC depending on the size of the metastasis, we also investigated a model that includes a size threshold for the smallest segment detected positive that would provide an overall positive diagnosis for the entire SLN. When the size threshold was 350 µm (Model 2), the area under the curve (AUC) increased to 0.93 [0.83–0.98]. For the same level of 80% sensitivity, this model achieved 96.97% specificity [95% CI 95.82–97.59%] (12 true positives, 64 true negatives, 3 false negative and 2 false positives).

The preliminary performance results for the AF-Raman analysis compare favourably with the performance of OSNA (~ 87% sensitivity and 92–98% specificity), technique that has been recommended by NICE [11]. Using Model 2 in a high specificity operating regime of 97% specificity, the AF-Raman provided 80% sensitivity, with only two samples out of 15 being false negative. Histology indicated that for these two samples the metastases were smaller than 2 mm (i.e., micro-metastases). According to the current NICE guidelines, only patients with one or more SLN macro-metastases (> 2 mm) would require ALNC [32]. Therefore, false negative diagnosis of SLNs with isolated metastatic cells or micro-metastases (between 0.2 and 2 mm) would not impact the treatment. However, one of the key disadvantages of OSNA and Metasin tests is the fact that they consume the LNs and therefore histology is compromised. While analysing only half of the node is possible, this leads to allocation bias. For this reason, the use of OSNA has been limited and Metasin has not been recommended by NICE [32]. On the other hand, the AF-Raman measurements are non-destructive and LNs are not damaged and can be analysed by histology afterwards. While the sensitivity of 80% is slightly lower than 87% for OSNA (at similar level of 97% specificity), the main cases of false positive AF-Raman detections in this study were caused by histiocytes. Histiocytes are randomly distributed throughout the tissue and identifying them in unstained tissue using AF imaging was difficult. Therefore, only few Raman spectra from this tissue type were recorded for the training set. Targeting and measuring more Raman spectra of histiocytes to re-train the Raman classification models may improve the discrimination between metastasis and normal LNs.

A substantial advantage of the AF-Raman technique is that the analysis provides a quantitative diagnosis image that requires no subjective interpretation by the user. Compared to other imaging techniques that rely on subjective decisions based on structural or morphological characteristics of tissue, quantitative techniques can reduce inter-user variability and minimise the time required for user training. The AF-Raman technique has been previously shown to produce reliable and repeatable results on skin tissue specimens, even when used by users with only few hours of training [33]. The AF-Raman measurement can also be fully automated by developing artificial intelligence algorithms to identify the LN and exclude the adipose tissue and change the microscope objectives between AF imaging and Raman spectroscopy. Such automated operation would require no input from the user once the measurement is started. Combined with the fact that AF-Raman does not require any sample preparation (fixation, micro-sectioning or staining), measurements can be started immediately after LN resection, by any member of the surgery team. The analysis is non-destructive, with no adverse effects on the tissue. Therefore, AF-Raman does not affect the standard histology evaluation of the samples, ensuring no interference with the current standards of care.

The overall measurement and analysis time for this prototype instrument varied between 20 to 30 min, which was considered acceptable for this proof-of-concept study. While this time is similar to other techniques, it could be significantly reduced by optimising the Raman spectrometer to reduce the acquisition time per spectrum. Previous studies using optimised Raman spectrometers reported acquisition times as short as 1 s per spectrum [34], compared to 5 s utilised here. Such instrument optimisation would reduce the total measurement and analysis time to only 5–10 min, which is significantly faster than frozen section histology. Further improvements could include the use of multi-foci Raman spectroscopy in power-sharing mode to measure Raman spectra from several locations simultaneously [35, 36]. Moreover, AF-Raman could also be used to determine the metastatic status of core biopsies from lymph nodes, rather than the entire lymph node. While a proof of principle study would be required to assess the feasibility of this application, AF-Raman measurement of core biopsies would be much faster due to the smaller size of the specimens. Typical core biopsies (2 × 10 mm^2^) could be investigated in less than 2 min, allowing identification of metastatic lymph nodes prior to excision. While much faster, this approach would not investigate the entire LN, which may result in missed metastases.

One of the limitations of this study is the relatively small sample size, with only 15 positive LNs included in the test set. This provides a rather wide confidence interval for the calculated sensitivity. Additionally, due to the small number of positive cases, the full range of types of lymph node metastasis could not be investigated (e.g., micro-metastatic lymph nodes or lymph nodes with isolated tumour cells). In order to obtain a more reliable estimation of detection accuracy for the AF-Raman instrument, a larger scale diagnostic test of accuracy would need to be performed, where there is no variation to the tissue processing of the specimens.

### Clinical application and relevance of the intraoperative spectroscopic evaluation of sentinel lymph nodes in current and future practice

De-escalation of axillary treatment has been evolving over the last decade. In most centres that follow UK NICE guidelines or NCCN guidelines, BC patients will not undergo axillary LN dissection if all the following criteria are present: cT1–T2, cN0, no preoperative chemotherapy, WBRT is planned and SLNs show 1–2 positive nodes (macrometastasis) on histological examination. Patients who do not fulfil these criteria will need to undergo axillary LN dissection. In some trials offering a radiotherapy to the axilla instead of ALNC did not affect the overall and disease-free survival. A recent review of ten years analysis for AMAROS trail showed a low axillary recurrence rate after both ALNC and axillary radiotherapy with no difference in overall and disease-free survival in patients with cT1-2, node-negative breast cancer and a positive sentinel node biopsy [9, 37]. This raises the argument whether identifying a positive SLN intraoperatively would have any meaningful clinical application in current practise. We recognise that axillary treatment in patients with positive SLN has been evolving recently. However, identifying a positive SLN intraoperatively is still relevant and important in certain circumstances. One of these scenarios is identifying a SLN following neoadjuvant chemotherapy. There is no evidence to suggest that omitting ALNC or offering radiotherapy to the axilla is sufficient in patients who underwent neoadjuvant chemotherapy and had a SLN postoperatively. ALNC is also recommended for cN0 patients with positive SLN and ineligible for IBCSG 23-01/Z0011/AMAROS/OTOASOR trials including BCS patients with > 2 SLN positive and for mastectomy patients with > 3 SLN positive. We also recognise that in many countries ALNC is still the preferred treatment for managing a positive SLN. The current use of frozen section for sentinel nodes in these countries has challenges including low sensitivity rate and lack of resources. Identifying patient with SLN who requires ALNC to benefit their clinical outcome requires a holistic analysis for all the trails that explore the axillary management.

Breast cancer adjuvant treatment is mainly influenced by the cancer biology rather than nodal status [38]. Nodal status is still important in adjuvant treatment in cases summarised by Reimer [38]: indication for regional node irradiation in ≥ pN2a disease, the decision of adding chemotherapy to endocrine treatment in luminal B tumors, the type and duration of endocrine treatment in hormone receptor-positive disease, adjuvant indication for dual anti-HER2 therapy, the post neo-/adjuvant indication for abemaciclib, considering monarchE data, and the postneoadjuvant indication for olaparib in patients with gBRCA mutation. The ability to identify SLN with high sensitivity and specificity intraoperatively will aid the treatment for the axilla in high-risk patients where there no sufficient data to support omitting ALNC by offering radiotherapy.

## Conclusions

This study demonstrates that the AF-Raman spectroscopy is a promising non-destructive technique for fast assessment of lymph nodes during breast cancer surgery. This technique exploits the main advantages of these two complementary techniques (speed for AF imaging, molecular specificity for Raman spectroscopy) and potentially could provide a practical solution to increase diagnosis accuracy of lymph node metastases. Furthermore, AF-Raman could potentially be adapted to determine the status of LNs infiltrated by other cancer types, by optimising the classification algorithms to specific tissue types.

### Supplementary Information

Below is the link to the electronic supplementary material.

Supplementary file1 (DOCX 1168 KB)

## Data Availability

The raw data (Raman spectra and AF images) and trained machine learning training data are not publicly available (potential IP protection) but are available from the corresponding author on reasonable request.
