# Supplementary material for: Intraoperative spectroscopic evaluation of sentinel lymph nodes in breast cancer surgery
Source: Breast Cancer Res Treat. 2024 May 20;207(1):223–32. doi: 10.1007/s10549-024-07349-z (PMC11230987; doi:10.1007/s10549-024-07349-z)
Supplement: Supplementary file 1 — Supplementary file1 (DOCX 1168 KB) [file 10549_2024_7349_MOESM1_ESM.docx]

**Supplementary information: Intraoperative spectroscopic evaluation of sentinel lymph nodes in breast cancer surgery**

**Processing of Raman spectra**

Because Raman raster-scanning required long acquisition times (1-2 hours), which interfered with the clinical pathway, the Raman maps were recorded from LN samples fixed in formaldehyde. However, one disadvantage of this approach is that fixation may cause biochemical changes in tissue that might affect the performance of the classification models when applied to fresh LN samples. Thus, the Raman bands which were affected by fixation were excluded from the spectral classification models. Raman spectra were pre-processed to remove cosmic ray spikes, background signals, and averaged using a 3 x 3 moving average filter. The signal to noise ratio (SNR) was calculated as the ratio of the intensity of the Raman band at 1450 cm^-1^ (C-H deformations) to the root mean square value of noise in the region between 1370-1410 cm^-1^, which contains no Raman bands. Spectra with a SNR below 10 were discarded. The regions of tissue analysed by Raman spectroscopy were then compared with the adjacent H&E-stained section in order to correlate each Raman spectrum with the tissue structures identified by the histologists. For the Raman classification model, a total of 4772 spectra were included from 60 patients: 3995 from normal lymphoid tissue and 817 from metastasis.

**Segmentation of AF images of lymphoid tissue**

Once LNs were separated from adipose tissue, the regions of the AF image corresponding to LN tissue were segmented using the Canny edge detection algorithm. This algorithm aimed to identify homogeneous tissue within the samples, which is characterized by a co-localised, similar AF intensity.

The edges between different tissue structures were identified (based on differences in AF intensity and morphology) and then the edge map was negated to produce a mask for the AF segments corresponding to homogenous regions. K-means clustering was performed to group adjoining segments that had the same relative AF intensity. The resulting AF segmentation map was then filtered to remove small segments generated by speckle noise or contaminants. Sampling points for Raman spectroscopy measurements were then allocated to each segment based on a uniform distribution.

Two parameters, ρ and ε, were calculated to evaluate the performance of the segmentation algorithms [29]. ρ is defined as the fraction of tumour detected by the segmentation algorithm, and ε represents the proportion of healthy tissue captured in segments containing metastasis. An ideal segmentation algorithm would retain the entire metastasis region (ρ=1), without any healthy tissue present in segments that contains metastasis (ε=0). This represents the maximal probability of Raman sampling points probing the tumour. The metastasis “hit rate” was calculated as the ratio of the number of Raman sampling points hitting the metastasis to the number of sampling points allocated to segments containing metastasis.

**Optimisation of the integrated AF-Raman analysis for LNs**

The first step in the workflow was the acquisition of the AF images of the biopsy area and then manual selection of the LN by the user. In the AF images, the surrounded adipose tissue was characterised by a higher AF intensity compared to lymphoid tissue and had by a mesh-like texture caused the higher AF intensity produced by the adipocyte nuclei and the lower intensity of their cytoplasm. Lymphoid tissue on the other hand appeared darker in the AF images and was surrounded by the capsule, which had high AF intensity due to its high collagen content (Figure S1).

AF images acquired at various magnifications (2x, 4x and 10x magnification objectives) were investigated to determine the efficiency of the segmentation algorithms to generate sampling points for Raman spectroscopy measurements (Figure S1). When applied to the AF images captured with the 2x and 4x objectives, the segmentation algorithm captured a median of 81% of the metastatic area observed in the H&E sections (median ρ=0.81), with a range of 73-86% and 76-86% for 2x and 4x objectives, respectively. When applied to AF images measured with the 10x magnification objective, the segmentation captured a median of 76% of the metastatic region (ρ=0.76, with a range of 0.64-0.82). No significant differences were found for other figures of merit when comparing different magnification objectives (see Figure S1). As the values of ρ were slightly higher for the 4x magnification, this objective was selected for the integrated AF-Raman measurements.

To increase the accuracy of detection for metastasis, an algorithm was developed to generate sampling points for Raman measurements based on the segmentation results. The proportion of Raman sampling points that were generated in the metastasis region of the lymph nodes (“hit-rate”) was 0.79 (79% of the metastasis area captured), regardless of the objective magnification. For the 4x magnification objective selected for the AF-Raman analysis, the range of the “hit-rate” was 0.53-0.94. These results predict that, for positive LN samples, the sampling points for Raman spectroscopy measurements would capture at least 53% of the metastasis area.


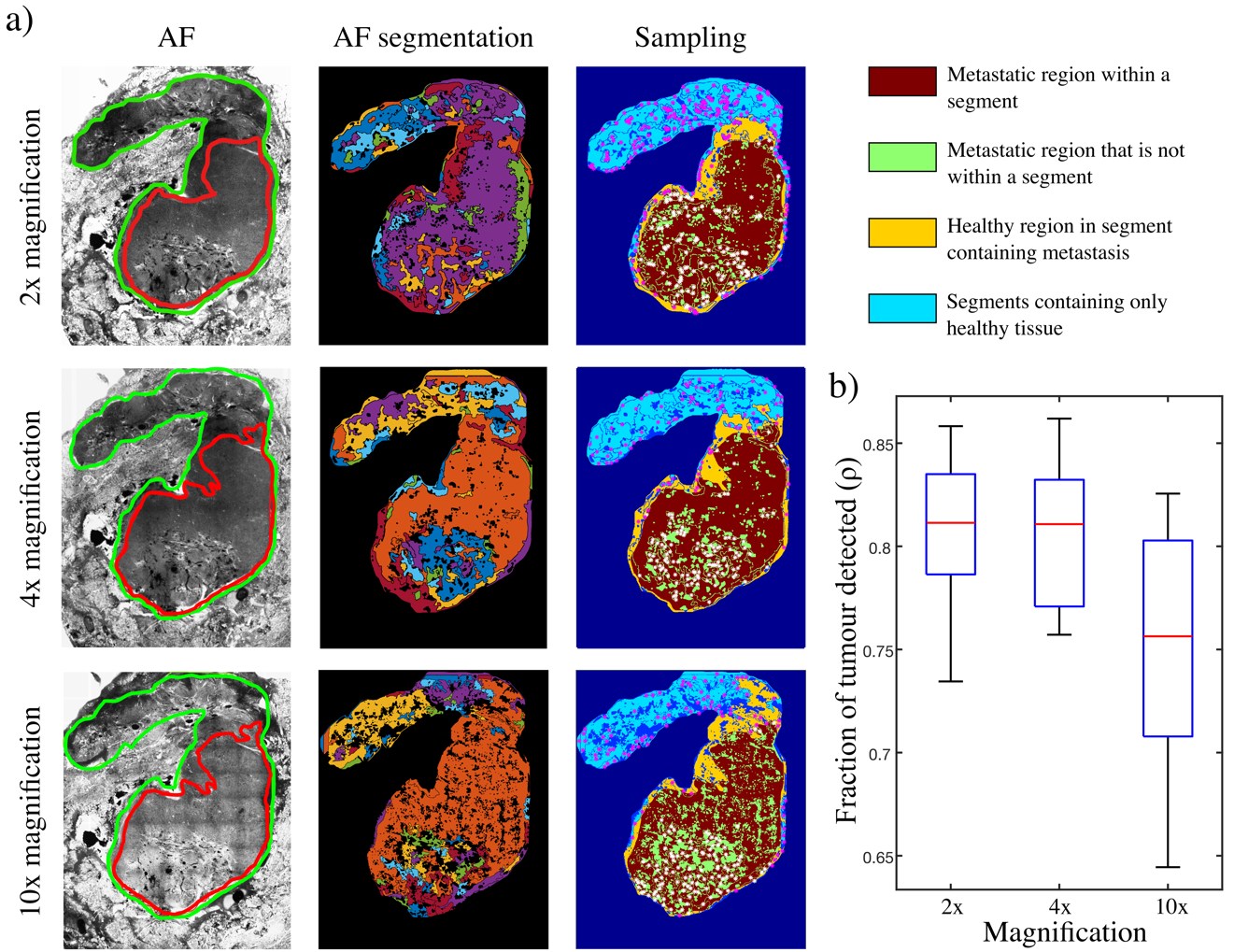


**Figure S1**. a) Segmentation of AF images of a lymph node containing metastasis acquired with 2x, 4x and 10x magnification objectives. The legend describes the colourmap in the "Sampling” images. AF images with lymphoid tissue annotated by a green outline and metastasis region annotated by a red outline; segmentation of the lymphoid tissue region; sampling points created for lymphoid tissue region. Red colour in sampling of AF image set indicates metastatic region within a segment, green represents metastatic region that is not within a segment (spilling area), yellow indicates healthy region in segment containing metastasis, blue indicates segments containing only healthy lymphoid tissue. b) The fraction of area corresponding to metastasis captured in segments (ρ) for all 14 positive LNs.


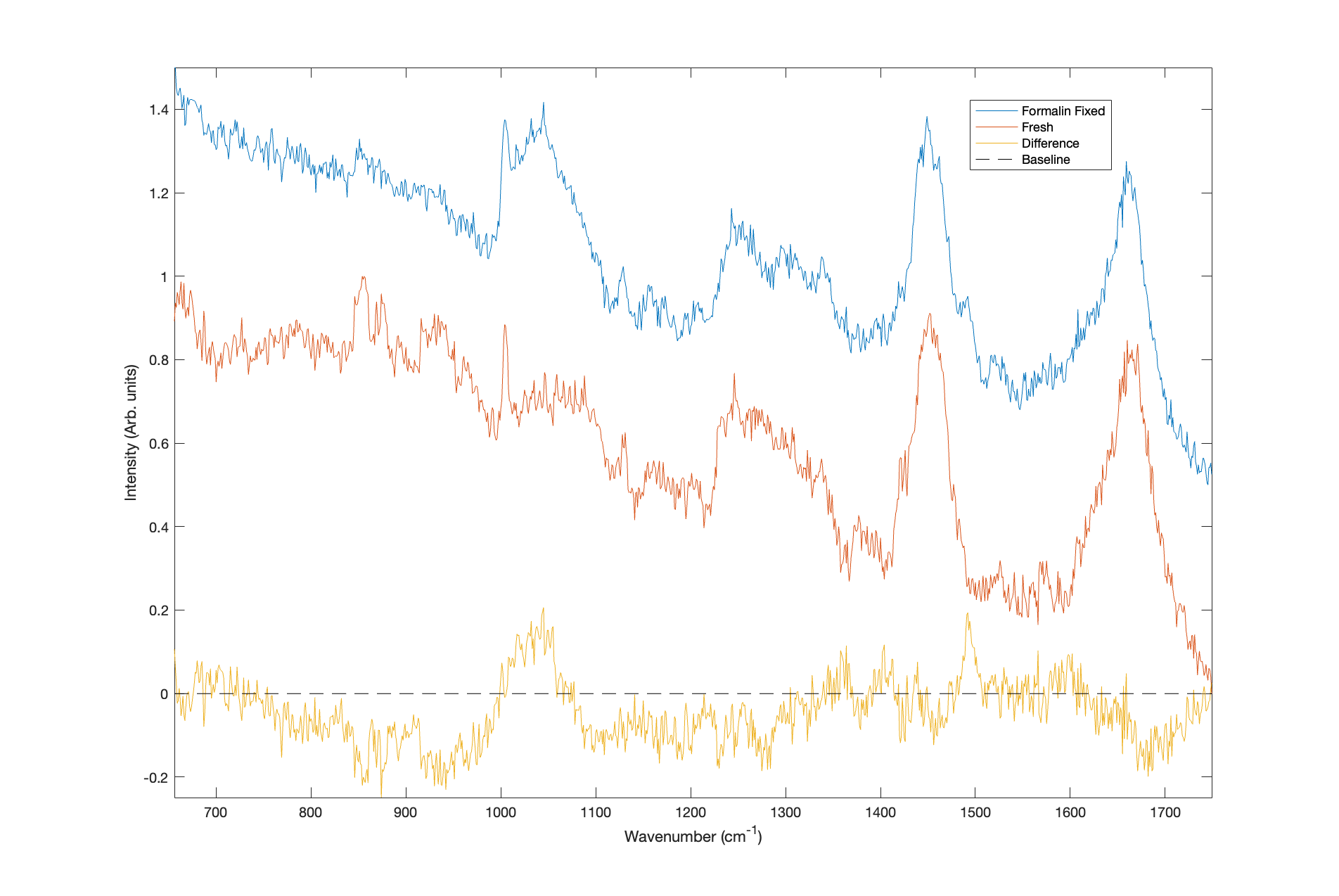


**Figure S2**. Single Raman spectra captured from metastatic regions, showing the difference between spectra captured from fixed (blue) and fresh (red) lymph nodes. The difference graph (yellow) shows that the Raman spectrum from the fixed specimen contains formalin specific bands (1000 – 1060 cm^-1^, 1490 cm^-1^), which are not present in the fresh spectrum. While still present in the fixed Raman spectrum, the spectral features between 750-1000 cm^-1^ and 1060-1300 cm^-1^ have a lower intensity than in the fresh spectrum.

**Table S1**: T-test detailing the statistical significance of the 10 spectral features used to train the Raman classification model. The t-test results show that nine out of the 10 features have statistically significant values between the metastasis and normal lymph node (LN) tissue (p-value<0.05). Feature 4 (850 cm^-1^) was still retained despite not showing a statistically significant discriminatory performance due to its observed prominence in metastasis Raman spectra. The t-test was performed on features from all metastatic spectra in the training set (616 spectra) and a random sampling of 616 normal lymph node tissue spectra from the training set.

| Raman feature | Wavenumber [cm^-1^] | Mean Metastasis | STD Metastasis | Mean Normal LN | STD Normal LN | p-value |
| --- | --- | --- | --- | --- | --- | --- |
| 1 | 698 | 1.36E-03 | 1.00E-03 | 2.08E-03 | 1.59E-03 | 1.55E-20 |
| 2 | 718 | 1.15E-02 | 3.72E-03 | 1.59E-02 | 5.44E-03 | 5.58E-53 |
| 3 | 784 | 8.39E-03 | 5.05E-03 | 2.02E-02 | 1.32E-02 | 6.44E-70 |
| 4 | 850 | 2.94E-02 | 7.13E-03 | 3.01E-02 | 1.20E-02 | 2.39E-01 |
| 5 | 873 | 8.96E-03 | 4.09E-03 | 6.61E-03 | 4.66E-03 | 5.00E-17 |
| 6 | 947 | 4.92E-03 | 2.64E-03 | 4.42E-03 | 3.27E-03 | 4.24E-03 |
| 7 | 1158 | 1.43E-02 | 5.30E-03 | 1.62E-02 | 1.58E-02 | 6.43E-03 |
| 8 | 1252 | 1.96E-01 | 1.06E-02 | 1.88E-01 | 1.46E-02 | 2.50E-24 |
| 9 | 1450 | 3.39E-01 | 2.55E-02 | 3.54E-01 | 2.84E-02 | 2.41E-25 |
| 10 | 1584 | 5.75E-03 | 2.55E-03 | 8.59E-03 | 3.78E-03 | 8.91E-41 |

**Table S2**: Optimisation of the Raman classification models based on 5-fold cross-validation. Each model was optimised individually by varying the spectral features and other specific parameters. The spectral features used for training were a subset of the 10 Raman bands presented in section 3.3. The uncertainties represent the standard deviations of the values obtained from different 5-fold splits.

| **Model Type** | **Sensitivity %** | **Specificity %** | **Specifications** |
| --- | --- | --- | --- |
| **Linear Discriminant Analysis (LDA)** | 80.7±0.8 | 81.0± 0.2 | 8 features; Target sensitivity: 90% |
| **Multinomial Logistic Regression (MNLR)** | 83.0±0.6 | 83.4± 0.2 | 10 features; Target sensitivity: 91% |
| **Artificial Neural Network (ANN)** | 86.0±0.2 | 84.6± 0.5 | 8 features; 15 neurons in hidden layer |
| **K-Nearest Neighbors (KNN)** | 87.1± 0.4 | 86.4± 0.3 | 8 features; Euclidean distance; Target sensitivity: 92% |
| **SVM** | 86.6± 0.1 | 85.8± 0.5 | 8 features; Kernel: RBF Target sensitivity: 90% |

**Validation Sample Size**

In this feasibility study the sample size was limited by the time available: the validation part was limited to 12 months, 10 months recruitment/measurements and 2 months for data analysis. During this period we expected ~80 patients to be recruited (a rate of ~2 samples per week observed during the development of the AF and Raman classification models). Based on ~20% rate of positive nodes, we expected 16 positive and 64 negative lymph nodes.

Using reported values of expected sensitivity and specificity and confidence intervals for competing technologies as a benchmark (e.g. OSNA sensitivity 87% (95% CI 81-91%) and specificity 92% (95% CI 86-95%) [18] we computed the following 95% confidence intervals for these performance values using the expected number of samples in our study: 90.96-92.94% for an expected 92.19% specificity and 83.06-90.19% for 87.5% sensitivity. As these calculated confidence intervals were similar to the values reported for OSNA [17], we considered the sample size acceptable for this feasibility study.

**Table S3**: Patient and disease information for the positive lymph nodes and the false positive cases (negative nodes diagnosed positive by the AF-Raman algorithm). NA= no resisual tumour post neoaduvant therapy neither in the lymph node nor in the tumour.

| **Sample** | **Fresh** | **Metastatic** | **Age** | **Invasive Tumour Size (cm)** | **Grade** | **T** | **P** | **M** | **Histol Tumour Type** | **ER Status** | **PgR Status** | **Final HER2 Status** |
| --- | --- | --- | --- | --- | --- | --- | --- | --- | --- | --- | --- | --- |
| 1 | No | Yes | 77 | 2.7 | 3 |  |  |  | NST | +ve | +ve | +ve |
| 2 | No | Yes | 65 | 0.8 | 1 | 2 | 2 | 1 | NST | +ve | +ve | -ve |
| 3 | No | Yes | 76 | 2.3 | 2 | 3 | 2 | 1 | Mixed NST & solid papillary | +ve | +ve | -ve |
| 4 | No | Yes | 62 | 7.8 | 2 | 3 | 2 | 1 | Mixed NST & classical lobular | +ve | +ve | -ve |
| 5 | No | Yes | 51 | 7 | 3 | 3 | 3 | 2 | NST | -ve | -ve | +ve |
| 6 | No | Yes | 62 | 3 | 3 | 3 | 2 | 2 | NST | -ve | -ve | +ve |
| 7 | No | No | 62 | 0.4 | 1 | 1 | 2 | 1 | Tubular | +ve | +ve | -ve |
| 8 | No | Yes | 44 | 7 | 1 | 1 | 2 | 1 | Tubular | +ve | +ve | -ve |
| 9 | No | No | 59 | 0.8 | 2 | 3 | 2 | 1 | NST | +ve | +ve | -ve |
| 10 | No | Yes | 53 | 3.2 | 2 | 3 | 3 | 1 | NST | +ve | +ve | -ve |
| 11 | No | Yes | 77 | 2.5 | 3 | 3 | 3 | 3 | Mixed lobular and ductal | -ve | -ve | -ve |
| 12 | Yes | No | 43 | 3.7 | 3 | 3 | 3 | 3 | NST | +ve | +ve | -ve |
| 13 | Yes | Yes | 62 | 1.2 | 2 | 2 | 3 | 1 | Mixed | +ve | +ve | -ve |
| 14 | No | Yes | 36 | NA | NA | NA | NA | NA | NA | -ve | -ve | -ve |
| 15 | Yes | No | 69 | 2.6 | 2 | 2 | 3 | 2 | NST | +ve | +ve | -ve |
| 16 | Yes | No | 34 | NA | NA | NA | NA | NA | NST | +ve | +ve | +ve |
| 17 | Yes | Yes | 78 | 0.4 | 2 | 3 | 2 | 1 | Mixed lobular and NST | +ve | +ve | -ve |
| 18 | Yes | Yes | 55 | 0.8 | 3 | 3 | 3 | 2 | Lobular carcinoma | +ve | -ve | -ve |
| 19 | No | Yes | 49 | 2.5 | 3 | 3 | 3 | 3 | NST | +ve | -ve | -ve |
| 20 | Yes | Yes | 48 | 4 | 2 | 3 | 2 | 1 | Classica lobular | +ve | +ve | -ve |
